# Initial Public Health Laboratory Response After Hurricane Maria — Puerto Rico, 2017

**DOI:** 10.15585/mmwr.mm6711a5

**Published:** 2018-03-23

**Authors:** Jeniffer Concepción-Acevedo, Anita Patel, Carolina Luna-Pinto, Rafael González Peña, Rosa Ivette Cuevas Ruiz, Héctor Rivera Arbolay, Mayra Toro, Carmen Deseda, Victor R. De Jesus, Efrain Ribot, Jennifer-Quiñones Gonzalez, Gouthami Rao, Alfonsina De Leon Salazar, Marisela Ansbro, Brunilís B. White, Margaret C. Hardy, Joaudimir Castro Georgi, Rita Stinnett, Alexandra M. Mercante, David Lowe, Haley Martin, Angela Starks, Beverly Metchock, Stephanie Johnston, Tracy Dalton, Olga Joglar, Cortney Stafford, Monica Youngblood, Katherine Klein, Stephen Lindstrom, LaShondra Berman, Renee Galloway, Ilana J. Schafer, Henry Walke, Robyn Stoddard, Robin Connelly, Elaine McCaffery, Marie-Claire Rowlinson, Stephen Soroka, Darin T. Tranquillo, Anne Gaynor, Chris Mangal, Kelly Wroblewski, Atis Muehlenbachs, Reynolds M. Salerno, Matthew Lozier, Brittany Sunshine, Craig Shapiro, Dale Rose, Renee Funk, Satish K. Pillai, Eduardo O’Neill

**Affiliations:** ^1^Division of Foodborne and Waterborne Diseases, National Center for Emerging and Zoonotic Infectious Diseases, CDC; ^2^Influenza Coordination Unit, National Center for Immunizations and Respiratory Diseases, CDC; ^3^Office of the Director, Office for State, Tribal, Local and Territorial Support, CDC; ^4^Puerto Rico Department of Health; ^5^Division of Laboratory Sciences, National Center for Environmental Health, CDC; ^6^Division of Select Agents and Toxins, Office of Public Health Preparedness and Response, CDC; ^7^Division of STD Prevention, National Center for HIV/AIDS, Viral Hepatitis, STD, and TB Prevention, CDC; ^8^Laboratory Leadership Service; ^9^Division of Viral Diseases, National Center for Immunizations and Respiratory Diseases, CDC; ^10^Division of High-Consequence Pathogens and Pathology, National Center for Emerging and Zoonotic Infectious Diseases, CDC; ^11^Division of Tuberculosis Elimination, National Center for HIV/AIDS, Viral Hepatitis, STD, and TB Prevention, CDC; ^12^Influenza Division, National Center for Immunizations and Respiratory Diseases, CDC; ^13^Georgia Public Health Laboratory; ^14^Virginia Department of Health; ^15^Florida Department of Health; ^16^Office of the Director, National Center for Emerging and Zoonotic Infectious Diseases, CDC; ^17^Association of Public Health Laboratories, Silver Spring, Maryland; ^18^Office of the Director, Office of Infectious Diseases, CDC; ^19^Division of Laboratory Systems, Center for Surveillance, Epidemiology, and Laboratory Services, CDC; ^20^Division of Vector-Borne Diseases, National Center for Emerging and Zoonotic Infectious Diseases, CDC; ^21^Division of Preparedness and Emerging Infections, National Center for Emerging and Zoonotic Infectious Diseases, CDC; ^22^Division of Emergency Operations, Office of Public Health Preparedness and Response, CDC.

Hurricane Maria made landfall in Puerto Rico on September 20, 2017, causing major damage to infrastructure and severely limiting access to potable water, electric power, transportation, and communications. Public services that were affected included operations of the Puerto Rico Department of Health (PRDOH), which provides critical laboratory testing and surveillance for diseases and other health hazards. PRDOH requested assistance from CDC for the restoration of laboratory infrastructure, surveillance capacity, and diagnostic testing for selected priority diseases, including influenza, rabies, leptospirosis, salmonellosis, and tuberculosis. PRDOH, CDC, and the Association of Public Health Laboratories (APHL) collaborated to conduct rapid needs assessments and, with assistance from the CDC Foundation, implement a temporary transport system for shipping samples from Puerto Rico to the continental United States for surveillance and diagnostic and confirmatory testing. This report describes the initial laboratory emergency response and engagement efforts among federal, state, and nongovernmental partners to reestablish public health laboratory services severely affected by Hurricane Maria. The implementation of a sample transport system allowed Puerto Rico to reinitiate priority infectious disease surveillance and laboratory testing for patient and public health interventions, while awaiting the rebuilding and reinstatement of PRDOH laboratory services.

Hurricane Maria caused an estimated $90 billion in damage (*1*) and profoundly affected the island’s 3.7 million inhabitants (*2*). The main PRDOH laboratory facility in San Juan and regional facilities located in Arecibo, Ponce, and Mayagüez municipalities were severely affected by the hurricane. PRDOH laboratories provide critical biologic and chemical laboratory testing activities certified under the Clinical Laboratory Improvement Amendments (CLIA). The destruction of the island’s electrical power grid (Figure 1) compounded the situation and rendered the PRDOH laboratory system unable to test for infectious diseases or detect environmental hazards. PRDOH identified repair of the public health laboratories as a major priority during the posthurricane response and requested assistance from CDC with clinical testing as well as with structural and safety assessments to help guide the restoration process.

**FIGURE 1:**
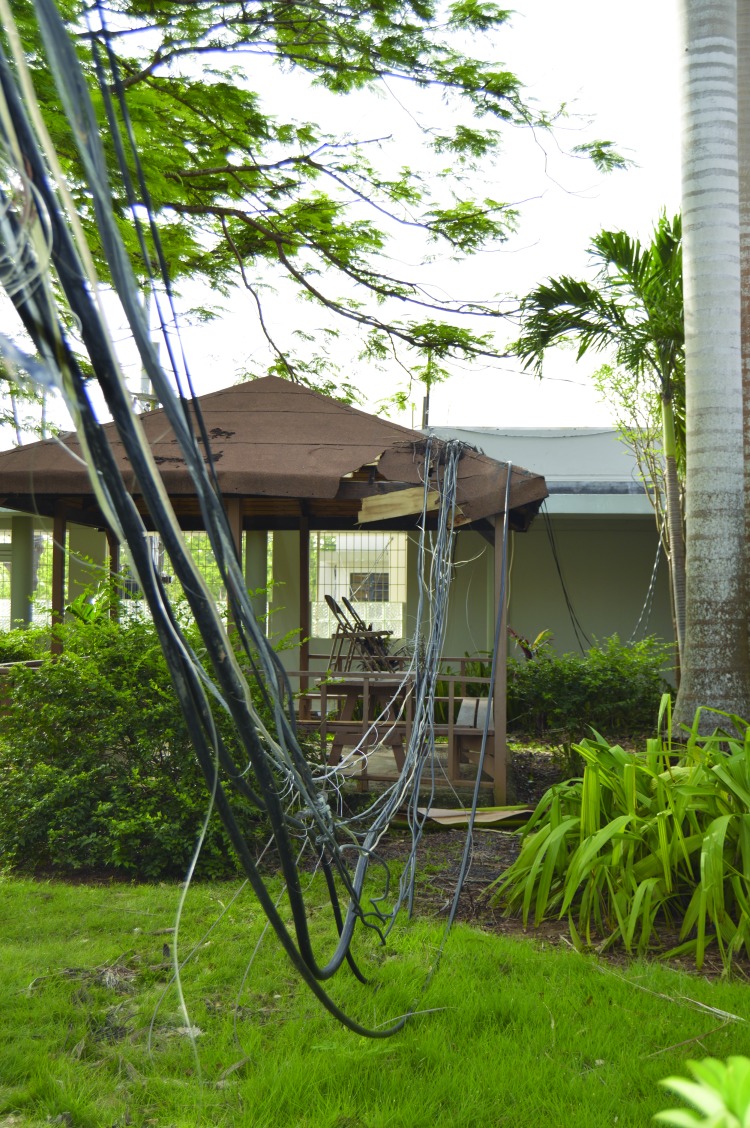
Disruption of electrical grid powering the Puerto Rico Department of Health laboratories caused by Hurricane Maria — San Juan, Puerto Rico, September 2017* Photo/CDC * The cables shown directly powered a section of the laboratory’s facility and were knocked down by the storm.

Federal, state, and nongovernmental partners collaborated to support and provide technical assistance to public health response activities in Puerto Rico. CDC field assignees in Puerto Rico were engaged in the effort, and laboratory scientists from Atlanta were deployed to Puerto Rico to help restore laboratory capacity for priority pathogens in the short term and to assist in the full restoration of PRDOH’s testing capacity in the long-term. As of February 2018, CDC had deployed 15 laboratory scientists to coordinate response and recovery activities and aid in the long-term restoration process. Partners collaborated to 1) conduct rapid laboratory needs assessments to understand the condition of laboratory facilities, prioritize activities to restore essential testing services, and determine long-term needs; 2) develop and implement a system for transporting samples from Puerto Rico to the continental United States for testing; and 3) establish an alternative, secure process for reporting testing results back to PRDOH. To coordinate sample shipments for testing at CDC or a state public health laboratory, the laboratory team partnered with APHL and the CDC Foundation, an independent nonprofit organization that supports CDC's health protection mission by mobilizing philanthropic and private sector resources.

On October 12, 2017, the first CDC laboratory team of two Spanish-speaking scientists was deployed to Puerto Rico; deployments continued, with teams rotating every 3 weeks. Deployed personnel had knowledge of laboratory facilities, testing operations, and CLIA requirements. Personnel with experience in laboratory management systems conducted laboratory assessments of the PRDOH facilities and recognized the urgent need to establish interim, alternative approaches to accomplish laboratory testing to guide surveillance and treatment management of priority diseases. The three core laboratory areas identified to have been affected by the hurricane were the electrical grid powering the facilities, the physical structure of laboratories, and equipment and reagents damaged by water leaks and power loss. Initial actions included requesting alternative power support through generators and procurement of equipment and reagents for each laboratory.

To address the immediate need for laboratory testing during the months after the hurricane, CDC and APHL worked with PRDOH to identify 16 CDC and state public health laboratories that could assist with clinical testing for selected diseases identified by the territorial epidemiologist as important for the local population; staff members streamlined shipping logistics and coordinated sample transport with the CDC Foundation. PRDOH provided guidance to health care facilities regarding priority diseases and requested that samples be sent to PRDOH for testing in continental United States laboratories. However, interruptions in communication limited the receipt of samples from the most affected rural municipalities. Transportation by couriers from some hospitals and clinics alleviated the transport limitations, and samples that were received were then accessioned at PRDOH laboratories and shipped to the continental United States. This centralized system permitted coordination and tracking of samples and test results by PRDOH.

Challenges in establishing this system included shipping companies’ inability to transport packages regularly to the continental United States. Affected carriers were unable to pick up packages directly from the PRDOH facility; these were transported to the shipping facility by deployed personnel, which resulted in delays. To ensure regular transport, sample shipments were delivered to the carrier facility Monday through Wednesday in the early morning. To replace water-damaged shipping containers, CDC sent containers to Puerto Rico with deployed personnel. APHL supplied additional shipping containers sent by carrier companies. When dry ice was available, it was picked up by deployed personnel from the only vendor operating under generator power on the island. The first package containing eight samples for tuberculosis testing using this transport system was sent to CDC on October 17, 2017 (Figure 2). As of January 27, 2018, PRDOH had shipped >1,700 samples using the transport system established by the laboratory team and partners, which resulted in the identification of nearly 350 cases of high-priority infectious diseases.

**FIGURE 2:**
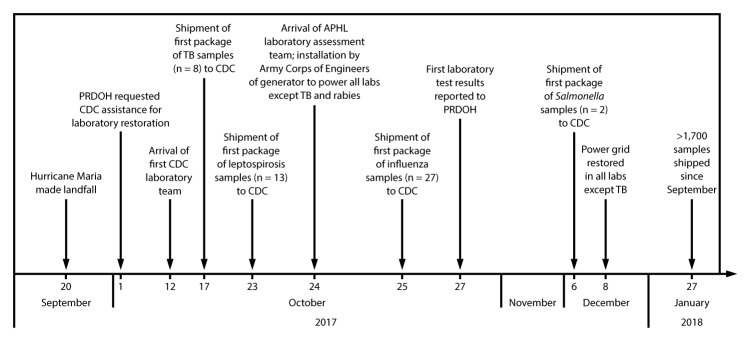
Restoration of laboratory services after Hurricane Maria — San Juan, Puerto Rico, October 2017–January 2018 **Abbreviations: **APHL = Association of Public Health Laboratories; PRDOH = Puerto Rico Department of Health; TB = tuberculosis.

In the immediate aftermath of the hurricane, PRDOH laboratories were relying on a single generator to power basic critical laboratory equipment and fax communication as the primary method for reporting clinical results. Because of the lack of reliable power in all laboratories, a secure file transfer protocol (FTP) site hosted at CDC in Atlanta was established for data exchange, which facilitated upload of results from reporting laboratories and retrieval by PRDOH from a computer with Internet access. The addition of a secure FTP site provided testing laboratories with multiple options for reporting clinical results including fax, encrypted email, and a secure portal. Most public health laboratories in San Juan were back on the power grid on December 8, 2017. In mid-December 2017, the Hurricane Maria response in Puerto Rico transitioned from response to recovery phase.

## Discussion

The establishment and implementation of a sample transport system allowed PRDOH to reestablish priority infectious disease testing and surveillance to guide patient and public health interventions, while awaiting the rebuilding and restoration of laboratory services. Alternative communication tools were critical for rapid reporting of high-priority infectious diseases. Laboratory assessments served as a basis for the development of a strategic framework for recovery efforts.

Laboratory recovery efforts will continue to focus on collaborating with PRDOH staff members to determine that 1) laboratory equipment receives and maintains required certification levels according to validation and quality assurance and quality control criteria, 2) the procurement of supplies, equipment, and other resources needed to restore clinical testing at PRDOH occurs, and 3) the identification of emerging needs is communicated and resolved.

With support from the CDC Foundation, deployed personnel continue to assist PRDOH staff members with sample transport logistics and are evaluating sample transport to test for additional pathogens until local testing can resume. CDC continues to provide laboratory support to PRDOH through expertise in quality systems, diagnostics, capacity building, laboratory risk assessments, and biosafety. These critical activities can serve as an example of successful federal, state, and nongovernmental partners’ collaboration to reestablish priority sample testing and disease surveillance for the 3.7 million residents of Puerto Rico.

**Summary**

**What is already known about this topic?**

Hurricane Maria devastated the U.S. territory of Puerto Rico in 2017, causing an estimated $90 billion in damage. As a result, the infrastructure and operations of the Puerto Rico Department of Health (PRDH) laboratories were severely disrupted, including diagnostic testing and disease surveillance for the island’s 3.7 million inhabitants.

**What is added by this report?**

CDC and partners established a system for temporary alternative testing for selected priority diseases in laboratories in the continental United States. Implementation of a sample transport system and establishment of alternative reporting methods allowed PRDH to reinitiate diagnostic testing and laboratory surveillance and helped guide patient and public health interventions.

**What are the implications for public health practice?**

Reestablishment of PRDH’s priority infectious disease testing and surveillance relied on collaboration and engagement among federal, state, and non-governmental partners, and the process can serve as a model for other jurisdictions facing public health emergencies. Incorporating practices described in this report in a preparedness plan might help in the rapid reestablishment of diagnostic testing and disease surveillance after a natural disaster.
